# Aging impairs deliberation and behavioral flexibility in inter-temporal choice

**DOI:** 10.3389/fnagi.2015.00041

**Published:** 2015-03-27

**Authors:** Yannick-André Breton, Kelsey D. Seeland, A. David Redish

**Affiliations:** Department of Neuroscience, University of MinnesotaMinneapolis, MN, USA

**Keywords:** rat, vicarious trial and error, delay-discounting, maze navigation, hippocampus, aging, spatial behavior

## Abstract

Inter-temporal choice depends on multiple, interacting systems, some of which may be compromised with age. Some of these systems may be responsible for ongoing trial-by-trial choice strategies. Some may represent the consequences of action. Some may be necessary for the coupling between anticipated consequences and strategies currently in use, flexibly guiding behavior. When faced with a difficult decision, rats will orient back and forth, a behavior termed “vicarious trial and error” (VTE). Recent experiments have linked the occurrence of VTE to hippocampal search processes and behavioral flexibility. We tested 5 month (*n* = 6), 9 month (*n* = 8) and over-27 month-old (*n* = 10) rats on a Spatial Adjusting Delay Discounting task to examine how aging impacted lap-by-lap strategies and VTE during inter-temporal choice. Rats chose between spatially separated food goals that provided a smaller-sooner or larger-later reward. On each lap, the delay to the larger-later reward was adjusted as a function of the rat's decisions, increasing by 1 s after delayed-side choices and decreasing by 1 s after non-delayed side choices. The strategies that aged rats used differed from those used in young and adult rats. Moreover, aged rats produced reliably more VTE behaviors, for protracted periods of time, uncoupled from behavioral flexibility.

## 1. Introduction

Senescence is associated with cognitive and neurobiological deficits. Human and non-human animals near the end of their lifespans show impaired spatial learning (Barnes, 1979; Gage et al., 1984), impaired reversal learning (Barense et al., 2002; Gallagher et al., 2011) and impaired working memory (Winocur and Moscovitch, 1990; Moscovitch and Winocur, 1995). Otherwise healthy aged rats take longer to learn to find a hidden platform in the Morris Water Maze and do not search for the platform in the vicinity of where it had been in probe trials, but show no impairments in locating the platform when it is visible (Gallagher et al., 1993). Aged rats also display impaired reversal learning on an odor discrimination task, but not the original discrimination of odors *per se* (Schoenbaum et al., 2002). Moreover, aged rats make more alternation errors in a T-maze that is baited with food on alternating sides, but make very few errors with respect to which side of the central stem is blocked with Plexiglas when it remains stationary across trials of a session (Ando and Ohashi, 1991). These results suggest impairments in spatial, but not cue learning; in reversal, but not discrimination learning; and in working, but not reference memory.

The cognitive impairments described above likely reflect neurobiological deficits acquired through the normal aging process. Barnes (1979) found aging-related attenuation in long-term potentiation and (Bondareff, 1979) found aging-related synaptic atrophy in the hippocampus, possibly underlying deficits in spatial learning. Neuroimaging studies of healthy older humans have suggested that prefrontal and orbitofrontal cortices are reduced compared to younger adults (Convit et al., 2001), possibly underlying deficits in reversal learning and working memory.

These cognitive and neurobiological impairments undoubtedly impact inter-temporal choice and decision-making. In procedures investigating the degree to which delayed rewards are discounted, animals are often provided the choice between two mutually exclusive goals: a smaller-sooner (SS) option, providing small immediate rewards, and a larger-later (LL) option, providing larger delayed rewards (Evenden and Ryan, 1996; Adriani and Laviola, 2003; Simon et al., 2010; Doremus-Fitzwater et al., 2012). Indifference between the two implies that the discounted value of the delayed reward is equal to the value of the non-delayed reward (Mazur, 1988). Increases in the magnitude of the LL reward increase the delay at which rats are indifferent between the two options. The rate of increase is reciprocally related to the degree to which delayed rewards are discounted: hyperbolic discounting predicts that the relation between the LL:SS amount ratio and the LL delay that produces indifference will be linear, and its slope will be proportional to the reciprocal of the discounting factor (Mazur and Biondi, 2009).

Often, the amount of food that the LL side provides remains constant, and a series of discrete LL delays are tested; group differences in impulsivity are then inferred from changes in the location of the delay-response function relating the proportion of LL choices to its delay. Delay durations on the LL side also tend to remain constant for multiple blocks of trials, following forced-choice trials establishing the operant-to-goal mapping. Performance on these tasks requires rats to have learned an association between an instrumental response and discounted reward value; that is, to win-stay. Moreover, the mapping of response and outcome remains consistent over multiple trials in a block, such that there is little uncertainty regarding the consequences of action. These blocked-trial tasks would therefore be insensitive to two possible consequences of the naturally aging brain. Aging may interfere with the type of strategy that rats use, as well as their ability to deliberate about the consequences of their choices.

Some researchers have proposed that hippocampal integrity is necessary for the maintenance of win-shift decision-making but not win-stay or conditioned place preference (McDonald and White, 1993). According to this hypothesis, the hippocampus is necessary for optimal performance when the “correct” response is dictated by a configuration of stimuli, such as the win-shift version of the 8-Arm Radial Maze Task. When the task required the animal to learn a reward-response pairing, such as in the win-stay version of the 8-Arm Radial Maze Task, or a Pavlovian stimulus-reward pairing, such as in the Conditioned Place Preference procedure, hippocampal lesions failed to impair performance. Given the hippocampal deficits associated with senescence, it is possible that aged rats would rely less on win-shift strategies and more on win-stay and Pavlovian strategies (Barnes, 1979).

The prefrontal cortex has also been strongly linked with task strategy. Wang et al. (2015) have proposed that deliberation about courses of action involves a circuit comprising prefrontal cortex and hippocampus. According to this hypothesis, the prefrontal cortex would send action plans to the hippocampus when the rat is uncertain about how to behave, while the hippocampus would simulate the outcomes of these plans. Given that aging has been associated with deficits in both structures, it is possible that aged rats would show not only different levels of deliberative behavior, but also a different coupling between deliberation and the type of decisions made.

**Figure 1:**
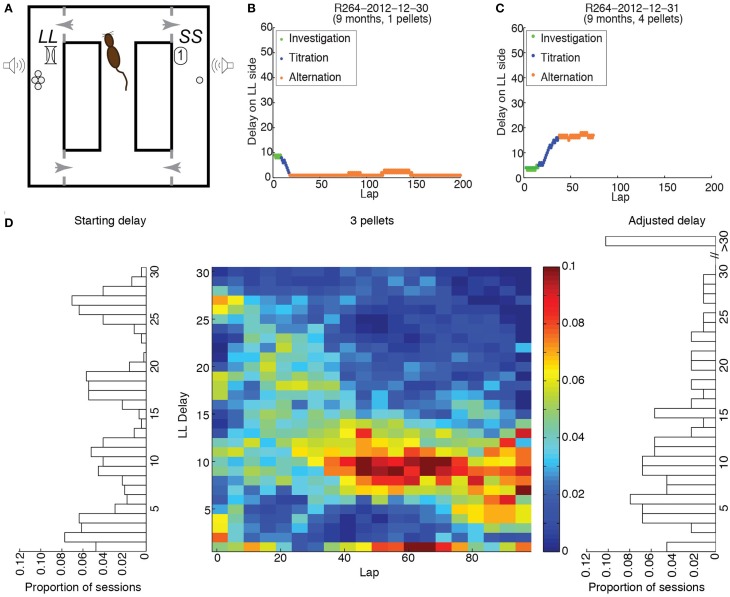
**Experimental procedure. (A)** Schematic of the maze. **(B)** Example of performance when the starting delay was larger than the compensatory delay; 1 pellet was delivered on the LL side. **(C)** Example of performance when the starting delay was lower than the compensatory delay; 4 pellets were delivered on the LL side. **(D)** Distributions of starting delays (left), final delays (right) and delays across laps (center) when 3 pellets were delivered on the LL side, for all rats.

We used the *Spatial Adjusting Delay Discounting Task* to investigate the impact of aging on strategy and deliberation during inter-temporal choice (Papale et al., 2012). In this task, delayed and immediate rewards are presented in spatially separate locations of a T-maze with a return path to the maze start (Figure 1). The larger-later (LL) side of the maze provides rewards after a delay that is adjusted by the rat's performance. The smaller-sooner (SS) side of the maze provides rewards after a trivial (1 s) delay. When the LL side is chosen, its attractiveness is decreased by increasing the delay to reward on the LL side by 1 s on the next lap. When the SS side is chosen, the attractiveness of the LL side increased by decreasing the delay to reward by 1 s on the next lap. The rat can maintain a constant delay on the LL side by alternating sides from lap to lap. Rats in this task make choices that drive the delay on the LL side to a duration that renders it as attractive as the SS side. Predominantly selecting one side implies a preference for the combination of reward and delay that can be expected there, while selecting both sides equally implies an indifference between the rewards and delays offered on both sides. By adjusting the delay on one side, we can identify which combinations of amounts and delays are equally preferred to a common small, non-delayed food reward. In the Spatially Adjusting Delay Discounting task, the rat's own choices determine how delays will be adjusted. In effect, the rat titrates the delay on one side to a duration such that the discounted value of the LL side is equivalent to the SS option.

Unlike neuroeconomic tasks presenting trials in blocks, which are susceptible to hysteretic and stability-induced distortions in performance (Cardinal et al., 2002; Breton et al., 2009), the nature of the task requires the rat to switch from repeating same-side (titration) choices to alternating side choices when both sides offer rewards of equal discounted value. If hippocampal integrity is necessary for maintenance of win-shift decision-making, and hippocampal function is impaired in aged animals, then it follows that aged rats would be more likely to win-stay. We predicted that this might result in differences in the degree to which delayed rewards are discounted.

When both the LL and SS sides provide a single food pellet, rats can use either a win-shift or a win-stay strategy. After titrating the delay on the LL side to the same duration as the SS side, a rat could alternate (win-shift) between the sides to maintain that delay, or could perseverate at the SS side (win-stay), as the LL side will not decrease below 1 s. When the LL side provides more food than the SS side, the LL delay that renders it equally attractive to the SS side will almost certainly be longer than the delay on the SS side. As a result, differences in discounted value that would arise from a win-stay strategy should drive the rat to pursue alternate side choices.

When faced with a difficult decision, rats will often pause and orient back and forth, a behavior identified as “vicarious trial and error” (VTE; Muenzinger and Gentry, 1931; Muenzinger, 1938; Tolman, 1948; Papale et al., 2012; Gardner et al., 2013; Schmidt et al., 2013). The qualitative appearance of VTE was likened in these early papers to “gestural thinking” and was hypothesized to reflect a deliberation over the potential consequences of the available actions. An impairment in this search process would induce impaired lap-by-lap strategies, as a rat without the ability to simulate the consequences of its actions would be unable to behave flexibly. Aged rats have well-known impairments in cognitive flexibility (Barense et al., 2002; Schoenbaum et al., 2002; Bizon et al., 2012). Moreover, aged rats have well-known hippocampal and prefrontal impairments (Barnes, 1979, 1994; Winocur and Moscovitch, 1990; Moscovitch and Winocur, 1995; Barnes et al., 1997; Small et al., 2011; Samson and Barnes, 2013), structures whose integrity alters VTE expression (Hu and Amsel, 1995; Griesbach et al., 1998; Blumenthal et al., 2011; Bett et al., 2012). It is therefore natural to presume that aged rats might express more VTE, or that they might engage in disrupted patterns of VTE behavior.

Since the delay to reward on the LL side depends on the rat's own performance, a forward search through imagined consequences of action is particularly useful early-on in the session, when rats need to anticipate the delays that will result from right and left choices based on previous choices and LL delays encountered (Papale et al., 2012; Stott and Redish, 2014). Once the rat is indifferent between the LL and SS sides, deliberation is not necessary on alternation laps, which maintain the delay constant. However, deliberation will be necessary on titration laps, since the delay on the LL side will change as a result (Papale et al., 2012; Stott and Redish, 2014).

The Spatial Adjusting Delay Discounting task provides a means of assessing the internally-driven coupling between VTE and behavioral flexibility across age groups. Rats have been shown to engage in VTE when task demands have not yet been automatized, early in the session, or when making adjustments to the delay after performance has been made automatic, late in the session (Johnson et al., 2007; Blumenthal et al., 2011; Steiner and Redish, 2012; Papale et al., 2012; Gardner et al., 2013; Schmidt et al., 2013). A contrast of the probability of a VTE event at different time points in the session, when the rat is engaged in different behavioral strategies, and the interaction between the two, permits us to evaluate the degree to which VTE is related to time alone, strategy alone, or behavioral flexibility in each age group.

We present here performance of young (5 months old), adult (9 months old) and aged (over 27 months old) rats on the Spatial Delay Discounting Task. Unlike previous reports, we used a maze with tall walls that prevented rats from orienting to the goal location directly. We used this task to identify (1) whether rats of all age groups would adjust delays on the LL side based on the amount of food that could be obtained there, (2) age- and reward size-related differences in trial-by-trial strategies, (3) age-related differences in the expression of VTE, and (4) age-related differences in the coupling of VTE to behavioral strategy at different points in the session.

## 2. Methods

### 2.1. Subjects

We used three ages of Fisher-Brown Norway (FBNF-1) rats to identify age-related differences in delay discounting and VTE: 5 months (5 mo., *n* = 6), 9 months (9 mo., *n* = 8), and older than 27 months (>27 mo., *n* = 10) old. Rats were individually housed in home cages in a temperature-controlled room, maintained at no less than 80% of their free-feeding body weight for the duration of the experiment, and provided *ad libitum* access to water in their home cages. Prior to beginning behavioral testing, rats were handled for 1 week and introduced to the plain food pellets that would be used as a reward in the experiment. Rats were run in six cycles of 1–2 animals per age group, ensuring age groups did not systematically differ with respect to handling and experimental parameters. All procedures were in accordance with National Institutes of Health guidelines for animal care and were approved by the Institutional Animal Care and Use Committee of the University of Minnesota.

### 2.2. Apparatus

Rats ran in mazes built from Duplo bricks (LEGO Group, Billund, Denmark) in two 97.8 × 97.8 cm arenas, arranged as in panel A of Figure 1. A 15.2 cm wide, 67.3 cm long central stem led to a choice point at which the rat could choose a left or right goal. Each goal was positioned along the outside wall of a 15.2 cm wide, 67.3 cm long aisle. Rats wore a red-LED backpack to locate them using a camera mounted above the maze. Speakers and food delivery ports were attached to the left and right outside walls of the maze. Med-Associates dispensers delivered 45 mg plain food pellets (TestDiet, Richmond, IN) via Nalgene tubing to the wall-mounted food delivery ports that constituted the left and right goal locations; food pellets were allowed to drop onto the maze track in the vicinity of the food port when a food reward was initiated. Experimental sessions were all controlled by in-house software written in MATLAB R2012a (The Mathworks, Natick, MA).

### 2.3. Procedure

#### 2.3.1. Door training

Although rats generally did not run backwards, to ensure they could not run backwards, the final 10 rats (5 months: *n* = 3; 9 months: *n* = 3; over-27 months: *n* = 4) ran in mazes containing one-way doors. The gray lines and arrows in panel A show the locations and allowed direction of movement of doors. Doors at the left and right ends of the choice point allowed unidirectional access to the food goals. Doors at the left and right ends opposite the choice point allowed unidirectional exits from the food goals.

The 10 rats using doors began a door-training phase after acclimation and initial exposure to the food pellet rewards, but before the 14-day habituation period. In this phase, a single wall partitioned a 97.8 × 97.8 cm testing arena into two chambers. One-way doors at each end of the central wall allowed rats to switch sides. In order to train these animals to use the one-way doors, rats were placed in one of the two chambers and food pellets were manually placed in the chamber not occupied by the rat. Rats could then push the appropriate door open with their snout, walk through, and eat the food pellets. The process was then repeated for the previously-occupied chamber, continuing in alternation for 40 min. Door training occurred for 5 days, prior to the habituation phase. Rats without doors began immediately in the experimental training phase described below. We found no differences between rats using doors and those who were manually blocked, so the data were pooled across all cycles of the experiment.

#### 2.3.2. Training

Following acclimation to the vivarium and initial exposure to the food pellet rewards used in the task, all rats underwent a 14 day habituation period in which one side of the maze was physically blocked with Duplo brick walls. We trained the rats in this phase to run down the central stem, turn left or right at the choice point, and obtain a single food pellet at the available goal after a 1 s delay. Each of these sessions lasted until the first of two conditions had been met: 200 (9 g) food pellets were delivered, or 45 min had elapsed.

After this initial habituation period, we removed the physical barriers, allowing access to both a *smaller-sooner* (SS) and *larger-later* (LL) side. A list of 28 combinations of delayed side (right or left), LL:SS pellet ratios (1, 2, 3, or 4) and starting delays (1–30) was generated. For the next 28 days, we selected the LL side, the number of food pellets delivered on the LL side, and the starting delay on the LL side from this randomized list. Each session lasted until the first of two conditions had been met: 200 (9 g) food pellets were delivered, or 90 min had elapsed. To ensure the data reflected stable performance after similar amounts of experience with the task, we analyze here the data obtained in the final 14 days. Age groups did not differ with respect to the number of pellets delivered on the LL side [χ^2^(2, *N* = 331) = 0.87, *p* = 0.65], with respect to the duration of the starting delay on the LL side [χ^2^(2, *N* = 331) = 0.35, *p* = 0.84], nor with respect to the sign of the difference between compensatory and starting delays [χ^2^(2, *N* = 331) = 2.2, *p* = 0.33].

#### 2.3.3. Spatial delay discounting task

Panel A of Figure 1 provides a schematic of the Spatial Delay Discounting task. After traveling down the central stem, the rat had to select either left or right food goals. Selecting the LL side increased the delay on that side by 1 s on the subsequent lap. Selecting the SS side decreased the delay on the LL side by 1 s on the subsequent lap. The SS side always delivered one food pellet after a 1-s delay. The LL side always delivered the same number of pellets within a day, but the number of pellets, starting delay, and left/right location of the LL side varied between days.

When a rat's backpack LED was within 12 cm of the food delivery port, the in-maze speakers constructed from disassembled headphones began sounding countdown tones of decreasing frequency. Tone frequencies were set with decrements of 250 Hz representing each second of delay. A 1 kHz tone signaled the delivery of a food reward.

Panels B and C of Figure 1 provide examples of performance on the Spatial Delay Discounting task. When a session has begun, the rat does not know the physical location, the number of pellets nor the duration of the delay for the LL side. Since the rat needs to investigate both sides to acquire the information it needs to make an informed decision, it should sample each side in alternation when the experimental session has begun, thereby maintaining the delay constant. Following this investigation phase, the rat should predominantly select one side, thereby titrating the LL delay upwards (B) or downwards (C). In both cases, the delay/amount combination that rendered the LL side as desirable as the SS side should have resulted in alternation between the two sides for the remainder of the experimental session.

### 2.4. Dependent measures

We assessed three dependent measures of performance on the task. To assess the delay that rendered the LL and SS equally valuable, we averaged the delay on the LL side across the last 20 laps of each session. We term this the adjusted delay. The adjusted delay represented a delay length that made the rat indifferent between the SS and LL sides. Hyperbolic discounting predicts that the delay at which the LL and SS sides are equally valued will be a linear function of the LL:SS pellet ratio (Mazur and Biondi, 2009), whereby the slope of this line is related to the reciprocal of the degree to which delayed rewards are discounted. Thus, we assessed the degree of delay discounting by determining, for each LL:SS pellet ratio, the adjusted delay.

To determine whether age groups differed with respect to win-shift and win-stay strategies, we identified laps on which rats alternated from the last chosen side (a win-shift strategy) and laps on which rats titrated the delay by repeating their last choice (a win-stay strategy). We then measured the proportion of alternation laps in entire sessions, or in 5-lap bins, to investigate whether age groups used these strategies differently on both whole-session and trial-by-trial levels.

To determine whether age groups differed with respect to vicarious trial-and-error behaviors, we measured the log-integrated absolute head velocity, z-scored by rat (*Z*[*Log*_10_[*Id*ϕ]]), from halfway down the central stem to the exit of the choice zone, where the rat would go through a door (if there were any) or where doors would have been (if there were none). Head position was identified in each frame of the 30 fps recorded video of the session with in-house software programmed in MATLAB R2012a, and smoothed with a Gaussian window of 0.2 s (σ = 0.1 s). Example videos of rats performing VTE and non-VTE laps, along with the inferred head position, have been provided for each age group in the online Supplementary Materials for this article.

We have previously reported that large *Z*[*Log*_10_[*Id*ϕ]] values indicate likely VTE passes, matching qualitative scoring of VTE (Papale et al., 2012). To quantify the probability of VTE, we fit a Gaussian mixture model to an age group's *Z*[*Log*_10_[*Id*ϕ]] values by an expectation-maximization algorithm implemented with MATLAB's *gmdistribution* tools, using 1–5 mixture components. We chose the mixture model for each group that had the smallest number of components and for which a Kolmogorov-Smirnov test of the data with the fit distribution was not statistically significant. The highest-mean component of that mixture was deemed to represent VTE, and the posterior probability of that component for a lap's *Z*[*Log*_10_[*Id*ϕ]] score provided the probability that it was a VTE event. We then measured the mean posterior probability of the VTE-related component in entire sessions, or on a lap-by-lap basis, to investigate whether age groups differed in their likelihood of performing VTE behaviors on both whole-session and trial-by-trial levels.

## 3. Results

### 3.1. Rats titrated LL delays to the LL:SS pellet ratio

To determine whether rats across age groups titrated the delays on the LL side to a consistent value, we performed two-sample Kolmogorov-Smirnov tests of the starting and adjusted delays for each LL:SS pellet ratio. For LL:SS pellet ratios of 1 through 3, we found significant differences between starting and adjusted delay distributions (KS-statistics: 0.7, 0.4, 0.2, respectively; *p* < 0.001, 0.001, 0.05, respectively). For LL:SS pellet ratios of 4, the Kolmogorov-Smirnov test revealed a trend toward significance (KS-statistic of 0.2, *p* = 0.068).

These data suggest that rats titrated the roughly uniform distribution of starting delays (left side of Figure 1D) to a consistent distribution of adjusted delays (right side of Figure 1D) for each LL:SS pellet ratio. The center panel of Figure 1 shows the distribution of delays on the LL side when it provided 3 pellets, across rats and sessions, in 5-lap bins for the first 100 laps. The appearance of the distribution of delays across laps shows that rats titrated the broad range of starting delays over the first 40 laps to a narrower range centered about 10.5 s, maintaining that LL delay over the next 60 laps.

**Figure 2:**
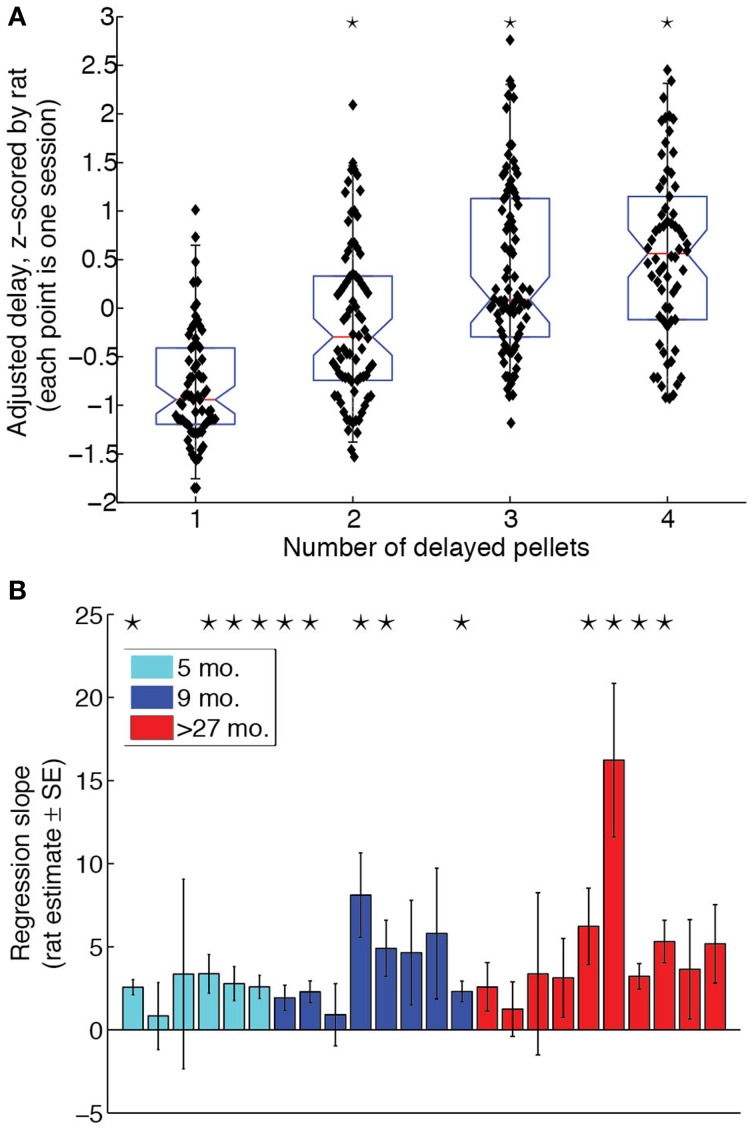
**Rats of all ages discounted delayed rewards. (A)** Adjusted delay, z-scored by rat, as a function of amount on LL side. Rats titrated to increasingly longer delays when the LL side delivered more pellets. Black stars indicate that the LL:SS pellet ratio significantly increased adjusting delays compared to all lower pellet ratios together. **(B)** Regression of adjusting delay on amount for each subject. Black stars indicate that the regression slope for a particular rat is statistically significant (*p* < 0.05). 4/6 of the 5 month-old (5 mo.), 5/8 of the 9 month-old (9 mo.), and 4/10 of the over-27 month-old (>27 mo.) rats had regression slopes significantly different from 0. All rats had positive regression slopes.

To determine whether rats titrated the LL delays to the LL:SS pellet ratio, we performed a One-Way ANOVA for the effect of pellet ratio on adjusted delays, z-scored by rat to eliminate any inter-subject variability. The ANOVA revealed a significant effect of pellet ratio [*F*_(3, 293)_ = 41.6, *p* < 0.0001, η^2^ = 0.29]. Orthogonal contrasts of pellet ratios with all lower pellet ratios revealed that increasing the pellet ratio significantly increased the adjusted delay for pellet ratios 2 through 4. The box plot in Figure 2A illustrates the effect of LL:SS pellet ratio on adjusted delays when eliminating inter-subject variability. A Two-Way (age × pellet ratio) ANOVA on raw adjusted delays revealed neither a main effect of age [*F*_(2, 21)_ = 2.7, *p* = 0.094, η^2^ = 0.04], nor an age × pellet ratio interaction [*F*_(6, 63)_ = 1.00, *p* = 0.435, η^2^ = 0.02], implying that groups did not differ in their overall willingness to wait nor in the degree to which delayed rewards were discounted. A linear regression revealed that the LL:SS pellet ratio was a significant linear predictor of raw adjusted delay [β = 3.65, *t*_(303)_ = 7.3, *p* < 0.0001, R^2^ = 0.28], as predicted by hyperbolic delay discounting (Mazur and Biondi, 2009). The slope of the regression of adjusted delay on pellet ratio was positive for all rats when done on a rat-by-rat basis. Figure 2B provides a bar graph of slope estimates and their standard errors for all animals, grouped by age. Although some rats did not show slopes that were significantly different from 0, groups did not differ with respect to proportion of significant linear regressions (*Z*-test for proportions performed for each pair of age groups: maximum *Z_prop_* = 1.0328, *p* = 0.151).

### 3.2. Lap-by-lap strategies changed across the lifespan

**Figure 3:**
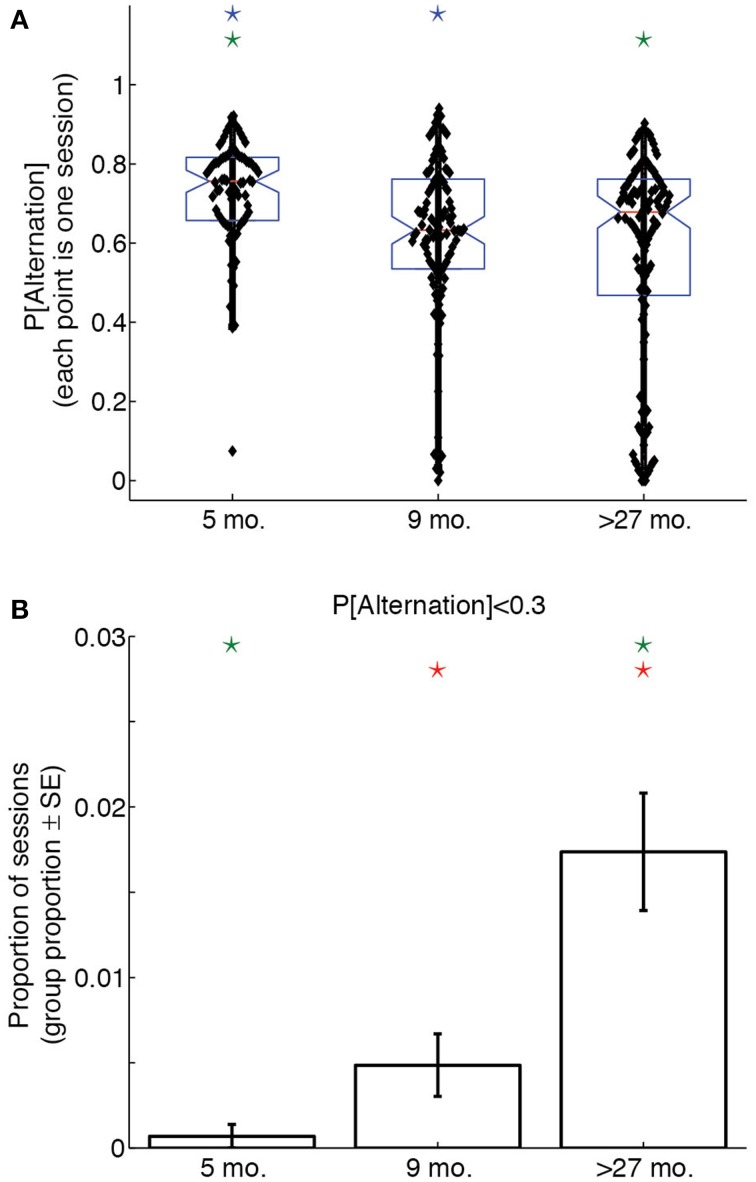
**Lap-by-lap strategies change across the lifespan. (A)** Proportion of alternation (win-shift) laps in a session as a function of age. 5 month-old rats alternate significantly more than 9 month-old or over-27 month-old rats. **(B)** Proportion of sessions in which rats alternated on fewer than 30% of trials. Over-27 month-old rats perseverate (win-stay) to a greater extent than 5 month-old or 9 month-old rats. Color-coded stars indicate significant group differences (*p* < 0.05, Bonferonni-corrected).

To assess whether age groups differed with respect to win-shift strategies throughout their experimental sessions, we calculated the proportion of laps on which rats alternated sides, thereby demonstrating a win-shift strategy, for each session. A One-Way ANOVA on overall session probabilities of alternation revealed a significant effect of age group [*F*_(2, 307)_ = 11.91, *p* < 0.0001, η^2^ = 0.07]. *Post-hoc* tests indicated that young rats performed more alternation than either adult or aged rats (*p* < 0.05, Bonferroni-corrected), suggesting 5 month-old rats used a win-shift strategy to a much greater extent than older rats. Figure 3A illustrates this difference with a box plot of the probability of alternation over the entire session for each age group.

To determine whether age groups differed with respect to perseverative behaviors throughout their experimental sessions, we calculated the proportion of sessions in each age group on which the overall probability of alternation was lower than 0.3. This probability of alternation corresponds to the lower limit of a 99.9% confidence interval about a null hypothesis distribution for which win-shift and win-stay strategies are equally likely over 100 laps. Alternating on fewer than 30% of laps would therefore represent a highly unlikely preponderance of same-side selections.

A Bonferroni-corrected series of pairwise Z-tests revealed that a significantly larger proportion of sessions from aged rats had alternation probabilities below 0.3 than young and adult rats (young vs. aged: *Z* = 3.2; adult vs. aged: *Z* = 4.3). Young and adult rats showed a trend toward significance (*Z* = 2.1). Figure 3B illustrates this difference: whereas young and adult rats had similarly low proportions of perseverative sessions, aged rats were much more likely to select the same side for over 70% of the laps of a session.

**Figure 4:**
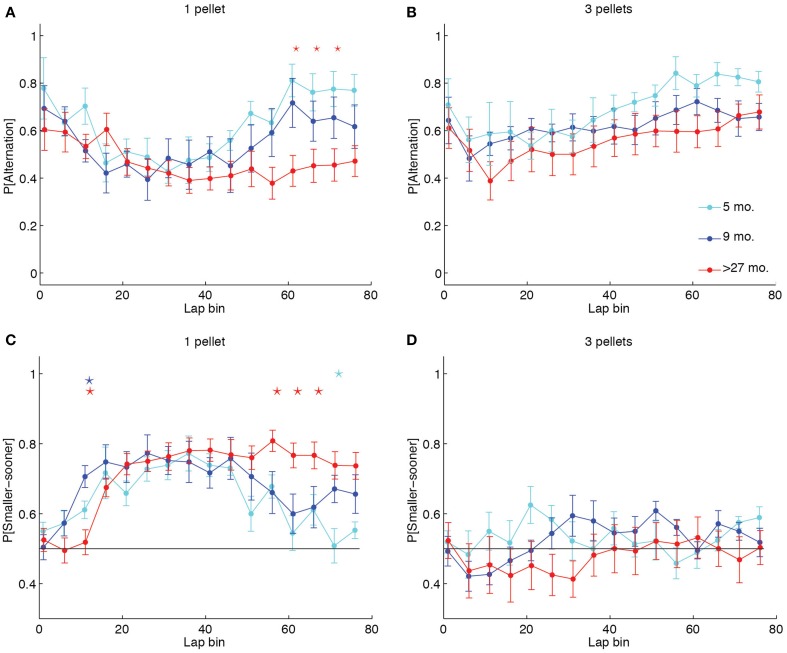
**Aged rats win-stay unless experimental contingencies force a win-shift strategy. (A, B)** Proportion of alternation laps in 5-lap bins when 1 or 3 pellets were delivered on the LL side. Five month-old and 9 month-old rats showed a pattern of alternation-titration-alternation when 1 pellet was delivered on the LL side. Over-27 month-old rats failed to return to an alternation pattern by the end of the session when both sides delivered 1 pellet. In contrast, when the LL:SS ratio was 3 pellets, all groups showed the alternation-titration-alternation pattern. **(C, D)** Proportion of SS side choices in 5-lap bins when 1 or 3 pellets were delivered on the LL side. Aged rats consistently chose the SS side when it delivered the same number of pellets as the LL side, but alternated when the two sides delivered differing amounts of food. Group-coded stars indicate a significant difference between the group and the mean of the other two in a particular lap bin (*p* < 0.05, Bonferroni-corrected).

To identify whether the increased likelihood of perseveration in aged rats was reflective of a different lap-by-lap strategy, we calculated the proportion of alternation laps over the first 80 laps in 5-lap bins for each LL:SS pellet ratio. We then conducted a Two-Way (age × lap) ANOVA on the mean probability of alternation across sessions for each pellet ratio. Whereas the ANOVAs revealed significant main effects of lap at all pellet ratios [1 pellet: *F*_(15, 315)_ = 5.77, *p* < 0.0001, η^2^ = 0.12; 2 pellets: *F*_(15, 315)_ = 2.57, *p* = 0.0012, η^2^ = 0.05; 3 pellets: *F*_(15, 314)_ = 6.22, *p* < 0.0001, η^2^ = 0.10; 4 pellet: *F*_(15, 304)_ = 2.69, *p* = 0.0007, η^2^ = 0.05], and no main effects of age at any pellet ratio, only when both sides delivered 1 pellet did the ANOVA reveal an age × lap interaction [*F*_(30, 315)_ = 1.79, *p* = 0.0083, η^2^ = 0.08] that would suggest an age difference in lap-by-lap strategy. Figure 4A illustrates this effect, plotting the mean probability of alternation in 5-lap bins for each age group when the LL side delivered 1 food pellet. Early-on, all groups were highly likely to alternate between options, presumably investigating the two sides' offers. Over time, all groups decreased their probability of alternation, presumably titrating the delay on the LL side to the number of pellets it delivered. Later in the session, however, over-27 month-old rats continued to show low levels of alternation, presumably perseverating at one side, whereas young and adult rats increased their levels of alternation. In contrast, Figure 4B illustrates the lap-by-lap pattern in strategy when the LL side delivered 3 pellets. In this case, all age groups showed a similar pattern of alternation across laps: high in the first 5, lower in subsequent laps, and high again later on in the session.

Perseveration at one side in the over-27 month-old animals could either result in repeated selections of the LL side, which would increase the delay indefinitely, or in repeated selections of the SS side, which would decrease the delay to a minimum of 1 s. To determine whether aged rats used an optimal win-stay strategy when both sides delivered equal amounts of food, we calculated the proportion of SS choices in 5-lap bins for each LL:SS pellet ratio. A series of Two-Way (age × lap) ANOVAs on the mean probability of SS choice at each pellet ratio reflected our findings on the probability of alternation: when both sides delivered one food pellet, aged rats predominantly selected the SS side, even late in the session when both sides delivered equal quantities of food after 1 s [*F*_(38, 399)_ = 2.89, *p* < 0.0001, η^2^ = 0.11], but when the LL side delivered more food, the lap-by-lap pattern of SS choices did not significantly depend on age [*F*_(38, 394)_ = 1.26, *p* = 0.1426, η^2^ = 0.05].

### 3.3. Vicarious trial-and-error had a different expression pattern over the lifespan

**Figure 5:**
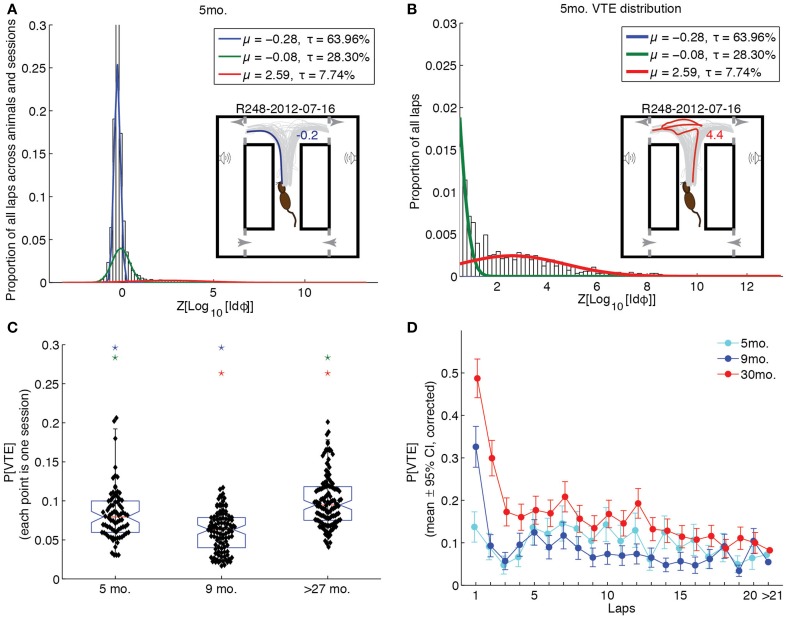
**Vicarious trial-and-error behaviors changed across the lifespan. (A)** Distribution of *Z*[*Log*_10_[*Id*ϕ]] values with Gaussian mixture model fit to 5 month-old rat data. Inset provides one session of passes through the choice point, with a representative example (blue) of a non-VTE lap. **(B)** Distribution of *Z*[*Log*_10_[*Id*ϕ]] values corresponding to VTE behaviors with Gaussian mixture model fit to 5 month-old rat data. Inset provides the same set of passes as A, with an example (red) of a VTE lap. **(C)** Probability of VTE as a function of age group. Color-coded stars indicate significant group differences. **(D)** Probability of VTE as a function of lap number across age groups. Non-overlapping error bars indicate a significant, Bonferroni-corrected group difference (*p* < 0.05). All groups are significantly different from each other on laps >21 (*p* < 0.05, Bonferroni-corrected).

To assess whether groups differed in their propensity for VTE behaviors, we fit a Gaussian mixture model to the log-integrated absolute head angle velocity, Z-scored by rat (*Z*[*Log*_10_[*Id*ϕ]]). The best-fitting model was that which had the fewest components for which a Kolmogorov-Smirnov test of the empirical *Z*[*Log*_10_[*Id*ϕ]] distribution against the fit distribution was not statistically significant. We thus chose the simplest mixture model that accounted for the data. For all age groups, 3 components were required to model the distribution of *Z*[*Log*_10_[*Id*ϕ]] values. Figure 5A shows the result of the fit overlain on the empirical distribution of *Z*[*Log*_10_[*Id*ϕ]] for the 5 month-old age group, along with an example of a non-VTE pass in the inset. Figure 5B shows this fit zoomed in on *Z*[*Log*_10_[*Id*ϕ]] values that putatively correspond to VTE (*Z*[*Log*_10_[*Id*ϕ]] ≥ 0.5), along with an example VTE pass in the inset.

The highest-mean component of each age group's Gaussian mixture model was deemed the VTE distribution for that group. We then calculated the probability of VTE for entire sessions by taking the mean posterior probability of the VTE distribution given *Z*[*Log*_10_[*Id*ϕ]] across laps. A One-Way ANOVA of the overall session probabilities of VTE revealed a significant effect of age group [*F*_(2, 307)_ = 44.03, *p* < 0.0001, η^2^ = 0.22]. Bonferroni-corrected *post-hoc* tests revealed that over-27 month-old rats were significantly more likely to produce VTE behaviors than either of the younger groups of rats (*p* < 0.05), and that 5 month-old rats were significantly more likely to produce VTE behaviors than 9 month-old rats. These results are illustrated by the box plots in Figure 5C.

To determine whether groups differed not only in overall levels of VTE, but in its expression over laps, we assessed the mean posterior probability of the VTE distribution for the first 20 laps, and laps 21 through the end, across sessions for each age group. A Two-Way (lap × age) ANOVA revealed significant main effects of lap [*F*_(19, 6169)_ = 10.88, *p* < 0.0001, η^2^ = 0.03] and age [*F*_(2, 6169)_ = 57.84, *p* < 0.0001, η^2^ = 0.02], and a significant lap × age interaction [*F*_(38, 6169)_ = 2.43, *p* < 0.0001, η^2^ = 0.01]. These results are plotted in Figure 5D. Over-27 month-old rats showed highest levels of VTE early-on that decreased over time [*F*_(19, 2671)_ = 10.5, *p* < 0.0001, η^2^ = 0.07], remaining high overall. 9 month-old rats also showed this pattern of decreasing likelihood of VTE [*F*_(19, 2109)_ = 7.34, *p* < 0.0001, η^2^ = 0.06], but decreased very quickly to low levels over the first few laps. In contrast, young rats did not show this quickly decreasing pattern over laps [*F*_(19, 1408)_ = 1.1, *p* = 0.35, η^2^ = 0.01], maintaining moderate levels of VTE throughout the first 20 laps of a session.

### 3.4. Vicarious trial-and-error was uncoupled from strategy in aged rats

The results above suggest that over-27 month-old rats engaged in VTE behaviors throughout sessions to a greater extent than 5 month-old and 9 month-old rats, and used different amount-dependent strategies than 5 month-old and 9 month-old rats to reach a common adjusted delay. Similarly, 5 month-old rats engaged in moderate amounts of VTE throughout sessions, and predominantly used a win-shift strategy at all LL:SS pellet ratios. We therefore sought to determine whether there were age-related differences in the coupling between lap-by-lap strategies (alternation or titration) and VTE behaviors, early in the session when performance had not yet been made automatic (first 25 laps) or late in the session when the adjusted delay had presumably been reached (last 25 laps).

**Figure 6:**
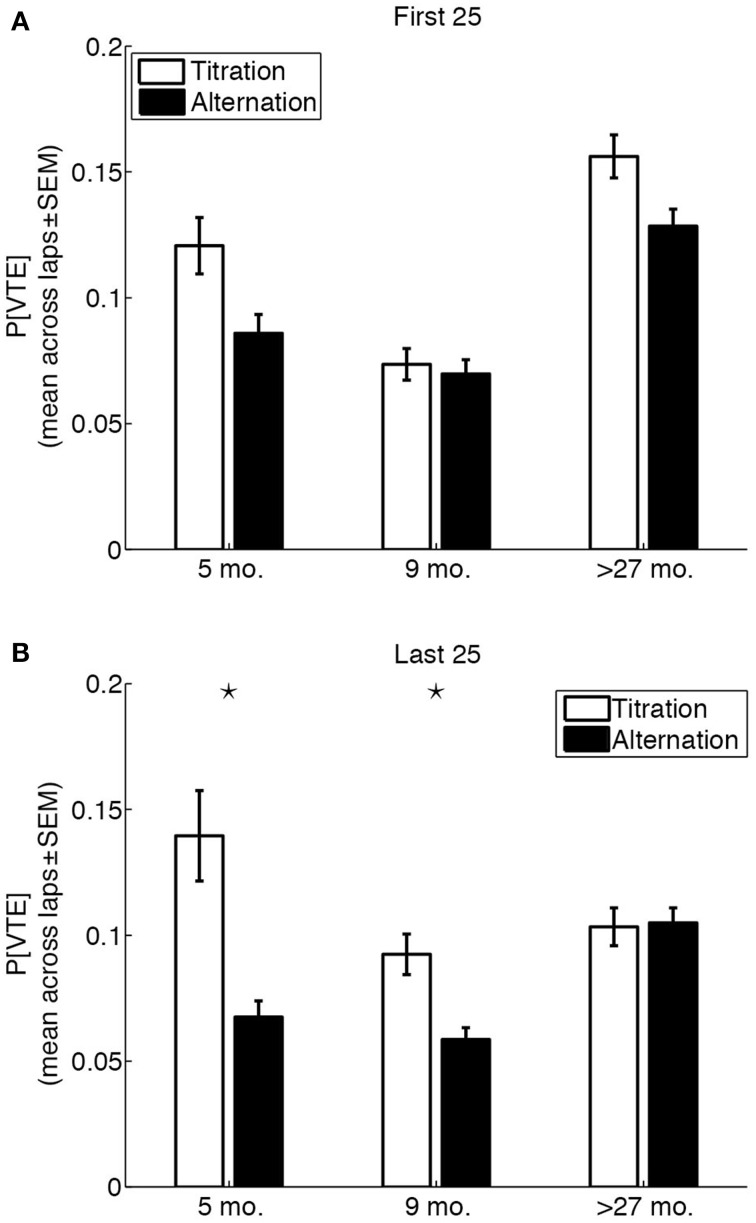
**Vicarious trial-and-error behaviors are uncoupled from strategy in aged rats. (A)** Probability of VTE in the first 25 laps of a session, as a function of rat age and strategy used on the lap. **(B)** Probability of of VTE in the last 25 laps of a session, as a function of rat age and strategy used on the lap. Black stars indicate significant titration/alternation differences (*p* < 0.05, Bonferroni-corrected).

An overall Three-Way (age × strategy × session phase) ANOVA on the probability of VTE revealed a significant Three-Way interaction [*F*_(2, 14,360)_ = 6.22, *p* = 0.002, η^2^ = 0.0008]. The ANOVA also revealed main effects of age [*F*_(2, 14,360)_ = 48.76, *p* <0.0001, η^2^ = 0.0068], strategy [*F*_(1, 14,360)_ = 37, *p* < 0.0001, η^2^ = 0.0026], and session phase [*F*_(1, 14,360)_ = 5.76, *p* = 0.0164, η^2^ = 0.0004]. Separate Two-Way (age × strategy) ANOVAs for the first and last 25 laps of a session revealed an interesting age-related change in the coupling between strategy and the probability of VTE (Figure 6). Whereas the age × strategy interaction was not statistically significant for the first 25 laps of a session [*F*_(2, 7402)_ = 2.18, *p* = 0.1126, η^2^ = 0.0005], the interaction was statistically significant for the last 25 laps of a session [*F*_(2, 6958)_ = 9.59, *p* < 0.0001, η^2^ = 0.003). *Post-hoc* tests revealed that differences in the probability of early VTE events between titration and alternation laps were not statistically significant for any of the three groups (*p* = 0.0072, 0.6514, 0.0101 for 5 month-old, 9 month-old and over-27 month-old rats, respectively; Bonferroni-corrected *p* = 7.6 × 10^−4^), and that only 5 month-old and 9 month-old rats showed a statistically significant difference in the probability of late VTE events between titration and alternation laps (*p* = 0.00001, 0.0001, 0.8801, for 5 month-old, 9 month-old and over-27 month-old rats, respectively; Bonferroni-corrected *p* = 7.6 × 10^−4^).

## 4. Discussion

Our results demonstrate that rats of all ages titrated the delay on the LL side to the amount of food it delivered on the Spatial Adjusting Delay Discounting Task. We replicated findings from Papale et al. (2012), who showed consistent, linear increases in adjusted delay with LL:SS pellet ratios of 1 through 4. This linear relationship was found on both the aggregate and rat-by-rat levels. Hyperbolic discounting predicts that the slope of the linear relationship between LL:SS pellet ratio and adjusted delay is reciprocally related to the delay discounting factor. Our results were consistent with this prediction, suggesting that the LL:SS pellet ratio is a significant linear predictor that accounts for 28% of the variance in adjusted delay. Although other groups have reported that both human and non-human animals discount delayed rewards less as they age (Mischel et al., 1989; Green et al., 1994, 1996; Barense et al., 2002; Adriani et al., 2004, 2010; Adriani and Laviola, 2006; Scheres et al., 2006; Olson et al., 2007; Steinberg et al., 2009; Whelan and McHugh, 2009; Romer et al., 2010; Simon et al., 2010; Prencipe et al., 2011; Doremus-Fitzwater et al., 2012; Roesch et al., 2012), we found no age group × pellet ratio interaction in adjusted delays on the aggregate level nor any group differences in the regression slope on the individual level that would be indicative of an age-induced decrease in the discounting of delayed rewards. While it is possible that we did not have the statistical power to detect an age difference in delay discounting, if it exists, our results suggest that a mere 2% of the variance in adjusted delay can be attributed to the age group × pellet ratio interaction.

Unlike many tests of inter-temporal discounting, our implementation of the Spatial Adjusting Delay Discounting Task required the rat to switch strategies when the LL:SS pellet ratio was greater than 1, but allowed the rat to use either a win-shift or a win-stay strategy when both sides provided a single pellet. When the LL side provided only 1 pellet, and the SS side provided 1 pellet after 1 s, the rat would optimally titrate the LL delay to 1 s by repeatedly selecting the (more attractive) SS side; however, once the two sides delivered equal amounts of food after an equal delay, the rat could continue to act optimally either by alternating between LL and SS sides (a win-shift strategy) or by continuing to select the SS side (a win-stay strategy), since the delay on the LL side reached a floor of 1 s. The above-described delay discounting results imply that for all LL:SS pellet ratios greater than 1, rats titrated the delay on the LL side to longer durations that were consistent across sessions. In order to maintain these durations, rats would have had to switch from a win-stay strategy on titration laps to a win-shift strategy on alternation laps. Our results suggest that young rats were more likely to alternate in experimental sessions, and that aged rats were more likely to perseverate throughout experimental sessions. Whereas young and adult rats showed a switch from win-stay to win-shift strategies for all LL:SS pellet ratios, aged rats continued to win-stay at the SS side when the LL:SS pellet ratio was 1. At greater LL:SS pellet ratios, aged rats showed a pattern similar to younger rats. In short, unless forced to alternate by experimental conditions, aged rats appeared to perseverate at the SS option.

In addition to its sensitivity to lap-by-lap strategies, our implementation of the Spatial Adjusting Delay Discounting task allowed us to assess deliberative behaviors in a maze that prevented rats from orienting directly to the left and right goals. Our quantitative analyses of the passes made through the choice point, normalized by rat, identified a mixture of three Gaussian distributions: two distributions with negative or near-zero means, and one distribution with a comparatively high mean greater than 2. Passes through the choice point that were likely to have come from this high-mean distribution qualitatively resembled what is traditionally categorized as a VTE event. The posterior probability of this high-mean distribution provided a proxy for the “VTE”-ness of a rat's pass through the choice point for each lap, on a readily understandable scale from 0 (definitely not VTE) to 1 (definitely VTE).

Our results suggest that the probability of VTE was age dependent overall, and lap dependent for each age group. Aged rats were more likely to produce VTE behaviors than young or adult rats, and young rats were more likely to produce VTE behaviors than adults. Interestingly, different patterns emerged when considering the probability of different age groups' VTE events across laps of a session. Adult rats produced VTE events in a pattern that is consistent with what (Papale et al., 2012) found using *Id*ϕ values Z-scored to each session, starting off high and decreasing to a minimum over laps. Young rats produced moderate levels of VTE that persisted over many laps. Aged rats, however, produced significantly more VTE events both early on and late, decreasing like younger adult rats over laps, but to a higher baseline level than young or adult rats.

Aged rats titrated delays on the LL side to equivalent levels, and showed statistically similar lap-by-lap strategies and side choices when the LL:SS pellet ratio was greater than 1. When both sides provided 1 food pellet, they used the perseverative win-stay strategy of repeatedly selecting the SS side. Despite these findings, aged rats appeared to produce more VTE overall, for a protracted period of time. In short, VTE was not helpful to these aged rats.

### 4.1. What is the role of VTE?

In younger rats, VTE was unrelated to behavioral strategy (alternation/titration) or session phase (first 25/last 25 laps) alone. Rather, higher-than-usual VTE occurred early in the session (within the first 10 laps) regardless of behavioral strategy, before task demands could have been automatized. Late in the session (in the final 25 laps), after task demands had presumably been automatized, VTE behaviors occurred to a significantly greater extent on titration laps. In contrast, aged rats showed more VTE behaviors on early laps, but no coupling between strategy and the expression of VTE on late laps.

Studies have shown that there are age-related hippocampal and prefrontal-cortical impairments (Barnes, 1979; Winocur and Moscovitch, 1990; Moscovitch and Winocur, 1995; Barnes et al., 1997; Barense et al., 2002; Gallagher et al., 2011; Samson and Barnes, 2013), and hippocampal integrity is necessary for the expression of VTE with simple decisions (Hu and Amsel, 1995; Griesbach et al., 1998; Blumenthal et al., 2011). Bett et al. (2012) found that, in a task requiring ongoing monitoring of reward contingencies, hippocampal lesions did not eliminate VTE; instead, lesions disrupted the relationship of VTE to behavioral flexibility, as we found in the aged rats. Why would the hippocampal atrophy that has been characterized in aging (Bondareff, 1979; Golomb et al., 1993; Van Petten, 2004) result in *greater* expression of VTE when decisions are ongoing and complex?

One proposal (Wang et al., 2015) is that the prefrontal cortex interacts with the hippocampus to covertly simulate potential outcomes of a decision. In this theory, the prefrontal cortex sends plans to the hippocampus when the course of action is unclear. The hippocampus then simulates those plans. The selected action is that which produces the most desirable simulated outcome. If no simulated outcome matches the desired goal with any certainty, the prefrontal cortex might continue to make requests to the hippocampus.

We propose that VTE occurs when the hippocampus runs through forward place sequences, simulating the results of each action plan. The simulations are used to train other decision-making systems early-on, when the rat is still uncertain about what to do. When other, faster decision-making systems are trained, the rat can rely on them rather than the slower simulation-based hippocampal system. If the rat needs to make changes, as would be necessary during late titration laps, the prefrontal cortex sends new requests for simulations to the hippocampus. Useful hippocampal simulations are necessary for the transition from deliberative to automatic systems of decision making.

On the Spatial Adjusting Delay Task, rats behave as though they know the reward contingencies (Papale et al., 2012). Titration laps alter the value of the LL side, which changes the relationship of the two goal locations, thereby requiring an update to the consequences of the potential action plans and updates to expectations of how each course of action will alter those consequences. Alternation, on the other hand, maintains the value of the two goal locations, which should neither require updates nor simulations late in the session. If VTE reflects the prefrontal request for simulation, and there is no need to choose among actions when alternating, the theory predicts that animals capable of useful simulations should only produce VTE behaviors on early laps, when the correct course of action is unclear, and on titration laps, when expectations need to be revised. Our analyses of young rats confirmed this prediction.

### 4.2. Conclusions

When associative, stimulus-stimulus information processing in the hippocampus is compromised, the hippocampal simulations will provide uninformative results. The uninformative simulations are then incapable of driving the transition from deliberative to non-deliberative decision-making systems. Consequently, animals with compromised hippocampi will continue to show VTE throughout the entire session, even though other decision making systems might be capable of directing behavior. Our results are consistent with this prediction: in aged rats with presumably compromised hippocampal and prefrontal processing, VTE was maintained throughout experimental sessions on all lap types. Our results suggest that the cognitive impairments seen in senescence may be the result of an impaired ability to vicariously try different plans when deliberating about the future, resulting in an increased reliance on decision-making systems that can operate more automatically.

### Conflict of interest statement

The authors declare that the research was conducted in the absence of any commercial or financial relationships that could be construed as a potential conflict of interest.
